# A Case Report of Successful Conservative Treatment for Infective Endocarditis Caused by *Gemella sanguinis*

**DOI:** 10.1155/2019/9382395

**Published:** 2019-01-02

**Authors:** Georgia Emmanouilidou, Panagiota Voukelatou, Ioannis Vrettos, Vasileia Aftzi, Konstantinos Dodos, Despoina Koumpouli, George Avgeropoulos, Andreas Kalliakmanis

**Affiliations:** ^1^2nd Department of Internal Medicine, General and Oncology Hospital of Kifissia “Agioi Anargyroi”, Noufaron and Timiou Stavrou 14, 14564 Athens, Greece; ^2^Clinical Laboratory Microbiology, General and Oncology Hospital of Kifissia “Agioi Anargyroi”, Noufaron and Timiou Stavrou 14, 14564 Athens, Greece; ^3^Department of Cardiology, General and Oncology Hospital of Kifissia “Agioi Anargyroi”, Noufaron and Timiou Stavrou 14, 14564 Athens, Greece

## Abstract

Infective endocarditis is defined as an infection of a native or prosthetic heart valve, the endocardial surface of the heart, or an indwelling cardiac device. Among the miscellaneous emerging opportunistic bacteria that can cause infective endocarditis is *Gemella sanguinis* that has been reported as a cause of infective endocarditis in nine cases in the past. All of the survivors received antimicrobial therapy and underwent prosthetic valve replacement surgery while, in general, a proportion of 40–50% of the patients with infective endocarditis underwent valve surgery. Our case illustrates that valve surgery, in combination with the administration of antibiotics, is not the only therapeutic option for infective endocarditis due to *Gemella sanguinis* and that a conservative management with prolonged administration of parenteral antibiotics under close supervision of the patient can be an option.

## 1. Introduction

Infective endocarditis is defined as an infection of a native or prosthetic heart valve, the endocardial surface of the heart, or an indwelling cardiac device [1]. Neurologic complications such as stroke are frequent, and they can be the presenting manifestations of the infection [2]. *Staphylococcus*, *Streptococcus*, *and Enterococcus* species are the most frequently isolated microorganisms, and they account for 80–90% of infective endocarditis cases [1]. Among the miscellaneous emerging opportunistic bacteria that can cause infective endocarditis is *Gemella* spp. [3]. These species are catalase-negative, Gram-positive cocci [4], and among them, *Gemella sanguinis* has been reported as a cause of infective endocarditis in nine cases [3, 5–12]. All of the survivors not only received antimicrobial therapy but also underwent valve replacement [3, 5–12].

In this report, we describe a case of *Gemella sanguinis* endocarditis manifested as stroke and treated successfully with only antibiotics.

## 2. Case Presentation

An 85-year-old female patient was admitted to our hospital with fever of 38°C, rigor, right hemiparesis with positive Babinski sign, and strabismus. Her medical history included heterozygous beta thalassemia, hypertension, and diabetes mellitus type 2. Physical examination revealed a blood pressure of 101/66 mmHg with a pulse rate of 81 beats/min and a grade II mitral valve pansystolic murmur. Basal rhonchi were noticed on lung auscultation. No other remarkable findings were revealed from the rest of the physical examination. The patient denied dental problems and had satisfactory oral hygiene. Complete blood count revealed a hematocrit  count of 32.6%, a hemoglobin count of 10.3 g/dl (12–16 g/dl), a red blood cell count of 5.36 M/*μ*l (3.9–5.6 M/*μ*l), total white blood cell count of 9.70K/*μ*l (4.0–11.0K/*μ*l) with 58% neutrophils, and a platelet count of 260K/*μ*l (150–400K/*μ*l). Her basal metabolic panel was normal, erythrocyte sedimentation rate was 52 mm/hr, and C-reactive protein levels were 11.50 mg/L (0.0–5.0 mg/L). Her glomerular filtration rate (GFR) was 65 ml/min. Urine and, by omission, only one blood culture was obtained. A brain computed tomography (CT) scan was performed, which excluded intracerebral hemorrhage. Initial treatment included ceftriaxone (2.0 g every day) and clindamycin (600 mg every 8 hours) for a possible aspiration pneumonia and acetylsalicylic acid (325 mg once daily). A second brain CT scan after 4 days revealed ischemic damages to the optic thalamus and the left cerebral hemisphere. A transthoracic echocardiogram revealed mild mitral and aortic regurgitation, a calcified mitral valve, and a mitral valve vegetation of 10 mm. The blood culture was positive for *Gemella sanguinis*. Gram stain of blood specimens showed Gram-positive cocci in pairs. The specimen in brain heart infusion (BHI) was incubated for 24 h and subcultured in blood agar. The colonies were described as nonhemolytic, tiny, pinpoint, smooth, and translucent to greyish. The organism was finally identified as *Gemella sanguinis* by a VITEK 2 Compact system. *Gemella sanguinis* identification via molecular methods was not available not only in the microbiological laboratory of our hospital but, as far as we know, also in the microbiological laboratories of several other hospitals. Furthermore, *Gemella sanguinis* is included in the VITEK 2 identification Gram-positive card (IGPC), but it is not included in the cards for antimicrobial susceptibility testing (AST) and thus an antibiogram was not feasible by means of this method. Unfortunately, an alternative method was not performed. Moreover, only one pair of blood cultures was taken at admission. However, the patient fulfilled one major (evidence of endocardial involvement) and three minor Duke's criteria (fever, ischemic stroke, and microbiological evidence of active infection with an organism consistent with endocarditis) for infective endocarditis. In the absence of an antimicrobial susceptibility testing for *Gemella sanguinis* and trying to avoid treatment failure, the treatment was switched, after 5 days, to vancomycin (1.0 g every 12 hours) and gentamicin (80 mg every 8 hours) according to her GFR. Further dental evaluation did not reveal dental lesions. The patient was monitored weekly with multiple transthoracic echocardiograms, and clinical assessment was performed daily in order to identify early the need for surgical intervention. After 1 month of treatment, the echocardiogram showed no definite mobile vegetation, and the patient was kept under conservative treatment. A transesophageal echocardiography after 6 weeks of treatment revealed mild mitral and aortic regurgitation with no definite vegetation or perivalvular abscess. The patient was discharged after having recovered fully.

## 3. Discussion

*Gemella* spp. belongs to a genus of Gram-positive, non-spore-forming, catalase-negative, oxidase-negative facultative anaerobes, forming single cells, pairs, or short chains. They are normal commensals of the oral cavity and the gastrointestinal tract [4, 6]. *Gemella sanguinis* was first described in 1998 [6] and has been reported once to be the cause of prosthetic joint infection [13] and nine times to be the cause of infective endocarditis [3, 5–12].

From the nine infective endocarditis cases caused by *Gemella sanguinis* that are published in the English literature, the first does not provide details about the patient and his treatment [6]. In five out of the eight remaining cases, endocarditis was associated with dental disease or previous valvular heart disease [7–11]. In our patient, there was no identifiable predisposing factor or source of infection. Her affected valve was the mitral valve while in the previous reports the aortic valve was affected in five cases [3, 8, 9, 11, 12], the mitral valve in two [7], and tricuspid valve in one [5]. Furthermore, in our case, the patient received intravenous antibiotics for a prolonged time, and the same treatment was also followed in 8 out of the 9 reported cases (for the remaining one, information is not available). One patient died because of intracerebral bleeding caused by a ruptured cerebral mycotic aneurysm [8]. In the other seven cases, all the patients needed prosthetic valve replacement surgery [3, 5, 7, 9–12]. Overall, 40–50% of patients with infective endocarditis underwent prosthetic valve replacement surgery [14]. There are three principal indications for valve surgery: heart failure caused by valvular regurgitation or obstruction, uncontrolled or complex infection, and for prevention of embolism. The objective of the surgery is to eradicate infection and reconstruct cardiac anatomy [1].

The fact that in all previously reported cases patients who survived needed surgery makes us consider that *Gemella sanguinis* is an aggressive and/or antibiotic-resistant microorganism, a fact that implies an indication for surgery [15]. However, in our case, daily clinical assessment for signs of heart failure and weekly echocardiographic examinations did not reveal complications that would necessitate surgical intervention.

Our case, the first reported in Greece and despite the limitation that the identification of *Gemella sanguinis* via molecular methods in a state or private lab was not feasible, illustrates that valve surgery, in combination with the administration of antibiotics, is not the only therapeutic option for infective endocarditis due to this microorganism. A conservative management with prolonged administration of parenteral antibiotics can be attained together with close supervision of the patient.
